# Predicting checkpoint inhibitor-associated autoimmune diabetes: prospects and limitations

**DOI:** 10.1172/JCI202477

**Published:** 2026-02-16

**Authors:** Kevan C. Herold, Ana Luisa Perdigoto

**Affiliations:** 1 Department of Internal Medicine, and; 2 Department of Immunobiology, Yale School of Medicine, New Haven, Connecticut, USA.; 3 Veterans Administration Hospital, West Haven, Connecticut, USA.

## Abstract

Checkpoint inhibitor–associated autoimmune diabetes (CIADM) is a life-altering and potentially life-threatening complication of immune checkpoint inhibitor (ICI) treatment in patients with cancer. Risk factors and predictors of this complication remain largely unknown. In this issue of the *JCI*, Wu et al. examined serum and PBMCs from 14 ICI-treated patients who developed CIADM and 28 matched controls. They identified several variables that were present prior to ICI treatment, including reduced pancreatic volume, islet autoantibodies, and biomarkers indicating immune cell activation, that together are highly predictive of development of CIADM. These findings could have profound clinical implications including treatment decisions, monitoring, and potential future prevention strategies.

## Introduction

Immune checkpoint inhibitors (ICIs) have revolutionized treatment of advanced malignancies, leading to improved survival for patients with a variety of cancers. However, ICIs can also result in immune-related adverse events (IRAEs) affecting most organs, including pancreatic islets, which can result in checkpoint inhibitor–associated autoimmune diabetes mellitus (CIADM) (1). With the use of ICIs expanding to more cancers and to the adjuvant and neoadjuvant setting, understanding mechanisms underlying IRAEs and potentially predicting their occurrence is becoming increasingly important.

Although similar to type 1 diabetes (T1D) in some ways, CIADM is distinct in its presentation and course. With an incidence of 0.2%–1.9% observed in patients treated with anti–programed death 1 (anti–PD-1) or anti–programed death ligand 1 (anti–PD-L1) antibodies alone or in combination with anti–cytotoxic T lymphocyte–associated protein 4 (anti-CTLA-4) antibody, CIADM is not common among the many patients with cancers treated with ICIs but can present acutely with severe hyperglycemia and, in many cases, diabetic ketoacidosis, a potentially life-threatening complication (1). Most patients have low or undetectable C-peptide levels indicative of rapid β cell destruction (2, 3). Autoantibodies, a key biomarker in diagnosing T1D, are found in fewer than half of patients with CIADM (2, 4). Treatment of CIADM is similar to T1D, with prompt initiation of insulin therapy. Like other endocrinopathies, CIADM is not reversible and can have a substantial effect on a patient’s quality of life, with the need for exogenous insulin and often labile control (5).

**Figure 1:**
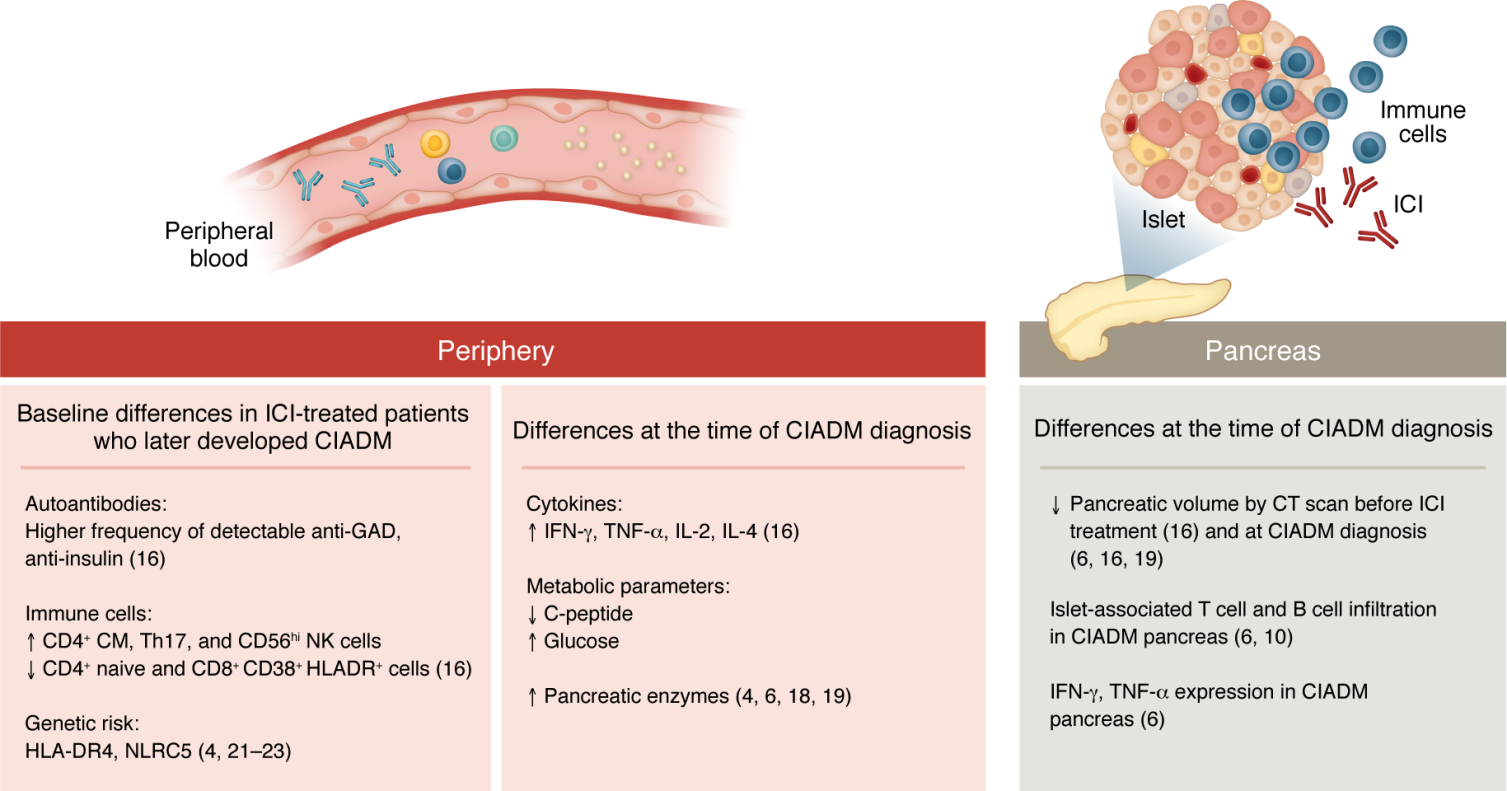
Mechanisms and predictors of CIADM in patients treated with ICIs. Prior studies in patients with CIADM suggest potential risk factors for development of CIADM, but these studies have been limited. Genetic risk factors include a higher prevalence of HLA-DR4 and a germline mutation in NLRC5, a key class I transcription activator, in patients with CIADM. Elevated pancreatic enzymes close to the time of CIADM diagnosis and reduced pancreatic volumes at the time of CIADM have been reported, consistent with pancreatic inflammation. In rare cases in which human pancreatic tissue is available, lymphocytic infiltrate and inflammatory cytokines have been reported. In this issue, Wu et al. (16) identified biomarkers in the periphery and changes in the pancreas at baseline (prior to ICI treatment) in patients who later developed CIADM compared with patients who did not. These included an increased frequency of detectable anti-GAD and anti-insulin antibodies, changes in immune cells by flow cytometry consistent with immune activation, and reduced pancreatic volume. Together, these baseline differences were predictive of the development of CIADM. Furthermore, Wu et al. identified elevated cytokine levels (IFN-γ, TNF-α, IL-2, IL-4) at the time of CIADM diagnosis that contribute to our understanding of the mechanisms underlying this complication. The figure summarizes the findings of Wu et al. as well as prior studies, as indicated by citation.

Our understanding of the mechanisms leading to CIADM are limited. Nonobese diabetic (NOD) mouse models treated with ICIs have demonstrated a role for CD8^+^ T cells (increased cytotoxic CD8^+^, terminally exhausted/effector-like CD8^+^, and reduced memory CD8^+^ T cells), CD4^+^ T cells (increased T-bet^hi^CD4^+^FoxP3^–^ and reduced memory CD4^+^FoxP3^–^ T cells), activated macrophages, and inflammatory cytokines such as IFN-γ and TNF-α in the pancreas of mice with CIADM (1, 6–8). A potential role for diabetogenic antigen-reactive CD8^+^ T cells in the blood and peripheral lymphoid organs was identified in NOD mice treated with ICIs (8, 9). In rare cases in which pancreatic tissue is available in patients with CIADM, lymphocytic infiltrate and IFN-γ and TNF-α expression have been observed (6, 10) (Figure 1).

Various risk factors and predictors of IRAEs have been proposed (11). These include HLA haplotypes, underlying autoimmune disease, baseline autoantibody levels, baseline/changes in cytokine levels, changes in T cells and B cells, and baseline microbiome composition, but they are not robust determinants (11–13). Potential risk factors of CIADM include prior spontaneous autoimmune thyroid disease or ICI-induced thyroid disease, high-risk T1D HLA haplotypes (i.e., HLA-DR4), younger age, and preexisting diabetes (1, 14, 15). In addition, the ICI is a determinant: CIADM most commonly occurs as a complication of anti–PD-1 or anti–PD-L1 treatment, particularly in combination with anti-CTLA-4.

## Immune changes predictive of CIADM

In this issue, Wu et al. identified biomarkers of CIADM development (16). They used a prospective biobank of data on 14 patients with metastatic melanoma who developed CIADM following ICI treatment and 28 matched controls who were also treated with ICI but did not develop CIADM. The authors analyzed several variables in serum and PBMC samples collected before treatment (baseline), upon ICI treatment (before CIADM development), and after development of CIADM. Pancreatic volume was also analyzed by CT volumetry. An important aspect of this study is the availability of prospective samples, which have been limited in CIADM. Wu et al. identified several baseline markers in patients with CIADM that together were found to be predictive of developing the complication (Figure 1).

Antibodies against glutamic acid decarboxylase (anti-GAD) and insulin (anti-insulin) were higher at baseline in patients with CIADM compared with controls. Of note, in most patients with CIADM, these autoantibodies remained in the normal range (generally defined as the 97.5th percentile of values that may be found in healthy individuals) but above detectable levels compared with controls (16). The elevated antibody levels are postulated to indicate an underlying β cell autoimmunity; however, in a study of individuals with adult-onset diabetes, Grace et al. found that antibodies against short GAD peptides were more predictive of early insulin requirement than were antibodies against the whole molecule (17). Thus, an increased autoantibody response may indicate activation of the pathogenic response. Other biomarkers examined by Wu et al. in the present work, including metabolic measures and changes in autoantibodies, were not predictive of CIADM.

We and others have reported reduced pancreatic volume and increased levels of pancreatic enzymes after ICI in patients who develop CIADM (4, 6, 18, 19). Here, Wu et al. confirmed the greater loss of pancreatic volume at CIADM diagnosis but also identified lower pancreatic volume in patients with CIADM compared with controls before treatment (16). Interestingly, a loss of pancreatic volume has also been observed in T1D, including prior to diabetes onset, suggesting that this may be a general finding in response to islet and/or pancreatic inflammation that is possibly related to reduced vascularity or a response to inflammatory mediators (20).

Wu et al.’s findings also suggest that baseline immune characteristics may identify those who are most likely to develop this adverse event. They identified evidence of an activated immune system, which involved a lower frequency of naive CD4^+^ T cells and an increased frequency of Th17 cells, CD4^+^ central memory cells, and CD56^hi^ NK cells. However, there was also a reduction in activated CD8^+^CD38^+^HLADR^+^ T cells, which might have been predicted to be effectors for the disease. Notably, similar changes in immune characteristics were observed when the authors compared patients who went on to develop any IRAE with patients who never developed a IRAE, suggesting that these changes may not be specific to CIADM and may warrant further investigation in other IRAEs (16).

The combined baseline changes point to underlying immunological and pancreatic changes that predispose patients to CIADM. By performing multiple logistic regression modeling using the baseline results of anti-GAD, anti-insulin, and pancreatic volume with or without flow cytometric analysis, they determined the receiver operating characteristic (ROC) performance of the models to be ROCs of greater than 0.96 and 0.89, respectively (16).

## Clinical implications, future directions, and challenges

Further validation of these findings is needed, and this study was limited to patients with metastatic melanoma, so studies to include a broader cancer population will be important to understand whether the findings are generalizable. Additional investigations of peripheral blood cells using single-cell transcriptomics might reveal even more specific markers that can identify risk. Furthermore, additional work will be needed to better delineate how Wu et al.’s proposed predictive model could be implemented clinically. The curious finding of elevated pancreatic enzymes, often without symptoms, prior to the development of CIADM, suggests that further investigations of pancreatic exocrine markers should be included in the modeling for prediction. Genetic risk factors may also be important determinants that could be identified prior to ICI treatment. The importance of HLA-DR4, which is increased in patients with CIADM, could not be evaluated in the study because of the limited number of participants (4, 21). In addition, our own work has identified a germline missense mutation (Pro191Leu) in the pattern recognition receptor NLRC5, a key class I transcription activator, in patients with CIADM compared with similarly treated patients, including those with other IRAEs, and the general population (22, 23). The finding by Wu et al. of activated CD4^+^ T cells suggests a possible mechanism, whereby T cell help can lead to activation of effector CD8^+^ T cells that may directly recognize β cell antigens in the islets. Inclusion of genetic data, such as HLA-DR4 and NLRC5 mutation status together, could potentially affect the ability to more accurately predict CIADM risk. Additional risk factors to include in larger studies may be those previously identified for IRAEs in general or CIADM specifically, such as a family history of diabetes or prior spontaneous or ICI-induced thyroid disease.

Much remains uncertain about the pathogenesis of CIADM as well as other IRAEs. Other studies have suggested a role for antigen-reactive T cells in CIADM, but their targets are not well defined in murine models or in patients (8, 9). As expected by their activity on effector T cells, Tregs, and other cells, the pathologic activity of immune cells differs with each ICI. In a recent issue of the *JCI*, Cakan et al. demonstrated an emergence of autoreactive mature naive B cells in patients treated with anti-CTLA-4 and anti–PD-1, but not anti–PD-1 alone (24). Thus, future analysis of risk may require characterizing immune cell subsets separately and specific to each ICI.

The ability to predict CIADM could have profound clinical implications and may enable preventative therapies if treatments that do not interfere with the antitumor efficacy of the cancer therapy were available. For example, the levels of IFN-γ, TNF-α, IL-2, and IL-4 in the serum were higher at the time of CIADM diagnosis, suggesting a role for these cytokines (16). Although this study focused on baseline changes and their predictive value, these findings contribute to our understanding of inflammatory mediators at the time of CIADM. IFN-γ and TNF-α have also been implicated in CIADM in mouse models (6, 7). Inhibition of IFN-γ or downstream signaling (JAK1/2) in mice (6, 9) and TNF-α blockade in humans (25) have shown promise as potential therapeutic targets for CIADM. These and other inflammatory cytokines, including those associated with the Th17 pathway, warrant further investigation. In select cases, knowing which patients are at higher risk for this complication could influence cancer treatment decisions if multiple options are available. Clinical monitoring for CIADM can also be personalized according to risk. At the present time, there are no clear guidelines as to how to monitor patients for development of CIADM. Maintaining residual β cell function in patients with T1D results in better glycemic control and reduced risk of hypoglycemia (26). Therefore, a tool that is predictive of CIADM and the possibility of using preventative interventions might change clinical practice.

The progress of cancer treatments with ICIs is nothing short of spectacular. The studies by Wu et al. and others lay the groundwork for prediction of IRAEs, shed light on immune mechanisms that may be relevant to tumor eradication, and suggest opportunities to improve the quality of life for those who benefit from the advances in treatments.

## Funding support

This work is the result of NIH funding, in whole or in part, and is subject to the NIH Public Access Policy. Through acceptance of this federal funding, the NIH has been given a right to make the work publicly available in PubMed Central.

- NIH grant K08CA282972 (to ALP).
- NIH grants R01DK057846 and R01CA227473 (to KCH).

## Version 1 02/16/2026

Electronic publication
